# Unilateral Resting Tremor in a Thigh Muscle in Parkinson’s Disease

**DOI:** 10.5334/tohm.556

**Published:** 2020-10-19

**Authors:** Sangmin Park, Ji-Hyun Choi, Won-Tae Yoon, Jee-Young Lee

**Affiliations:** 1 Department of Neurology, Seoul National University Boramae Hospital, Seoul, KR; 2 Department of Neurology, Kyungpook National University Chilgok Hospital, Daegu, KR; 3 Department of Neurology, Seoul National University Bundang Hospital, Seongnam, KR; 4 Department of Neurology, Kangbuk Samsung Hospital, Sungkyunkwan University School of Medicine, Seoul, KR; 5 Seoul National University College of Medicine, Seoul, KR

**Keywords:** Parkinson’s disease, resting tremor, thigh muscle

## Introduction

Resting tremor is the characteristic motor sign in Parkinson’s disease (PD), predominantly affecting the upper extremity, unilaterally. The rest tremor can appear in other body regions at 4–6 Hz frequencies [1,2]. It typically starts in the distal part of the upper extremity and then progresses proximally and to the other extremities over time [3]. However, isolated tremors in one lower limb can also be an initial manifestation of PD. Unilateral leg tremor in PD patients mostly involves the foot, and similar to those in upper extremities, it can evolve into thigh muscle resulting in a whole leg movement [4].

On the contrary to this steady rule of a distal-proximal gradient in parkinsonian resting tremor, we here to describe three patients with PD presented with unusual, isolated leg tremor starting from a thigh muscle without involving distal part of the leg, thereby extending our knowledge on the clinical features of parkinsonian resting tremor appearing in a leg.

## Case Series

### Case 1

A 57-year-old man presented with a slowly progressive leg tremor developed two years ago. He felt tremor in the proximal part of the right leg only with resting state. Initially, the rest tremor selectively appeared when he lay down in a supine position. Then, it also appeared in his right thigh when he lay down with a semi-supine position. (Video 1) or standing, but immediately disappeared after he started to walk. For the last three months, he felt rest tremor in his right hand as well. Despite evolving parkinsonian symptoms over the last three months, the leg tremor was still restricted to a thigh muscle. On initial examination, he showed bradykinesia and rigidity on his right side. Initial Movement Disorder Society-United PD Rating Scale (MDS-UPDRS) motor score was 15.5. Hoehn&Yahr (H&Y) stage was 1. Brain magnetic resonance imaging (MRI) showed no focal lesion. He started antiparkinsonian medication under the diagnosis of PD. His symptoms responded excellently to rasagiline 0.5 mg, levodopa 100 mg/carbidopa 25 mg 200 mg, trihexyphenidyl 3 mg daily, but mild degree rest tremor in a thigh muscle remained persistent until the latest follow-up 7 months.

**Video 1:** Tremor at rest appeared in his right thigh when he lay down with a semi-supine position. There is a tiny tremulous movement in the left leg, but this was not primary and was transmitted from the right leg movement.

### Case 2

A 59-year-old woman developed progressive left thigh tremor one month before the first clinic visit. She reported that the left thigh tremor specifically emerged only when she was lying on her back but with the knee flexed and did not experience leg tremors in other positions. On the initial neurological examination, there was mild bradykinesia in her left side. The left thigh tremor was explicitly observed while she was sitting with her legs crossed on the examination bed, and the tremor accentuated when she was distracted by doing repetitive finger tapping movement. (Video 2). As parkinsonian symptoms evolved over the last 20 months, the leg tremor was still restricted to a thigh muscle, but it only appeared when she was sitting her legs crossed and she was lying with knee flexion (a semi-supine position). The MDS-UPDRS motor score on the first exam was 6, and the H&Y stage was 1. Brain MRI showed no focal lesions. She was diagnosed as PD and showed an excellent response to rasagiline 1 mg, pramipexole extended-release 0.375 mg, and trihexyphenidyl 3 mg daily.

**Video 2:** Resting tremor in the left proximal thigh, especially hamstring muscle, appeared when she was sitting cross-legged and got worse with the distraction. The left great toe movement was not a tremor, and it was just transmitted from the body oscillation provoked by the right proximal leg tremor.

### Case 3

A 71-year-old woman developed rest tremor in her left thigh one year ago. The thigh tremor appeared when she posed a semi-supine position and was sitting her legs crossed. There was no resting tremor in other body parts. Neurological examination revealed left-sided mild bradykinesia and rigidity. The resting tremor was provoked in her left thigh while she was sitting cross-legged (Video 3). The MDS-UPDRS motor score on the first exam was 15, and the H&Y stage was 1. Brain MRI showed no focal lesion. Under the diagnosis of PD, she started rasagiline 0.5 mg, levodopa 100 mg/carbidopa 25 mg 150 mg daily, and showed excellent response in her bradykinesia. The leg tremor was only mildly improved.

**Video 3:** The resting tremor was provoked in her left thigh while she was sitting cross-legged.

### Dopamine transporter imaging

**Figure 1:**
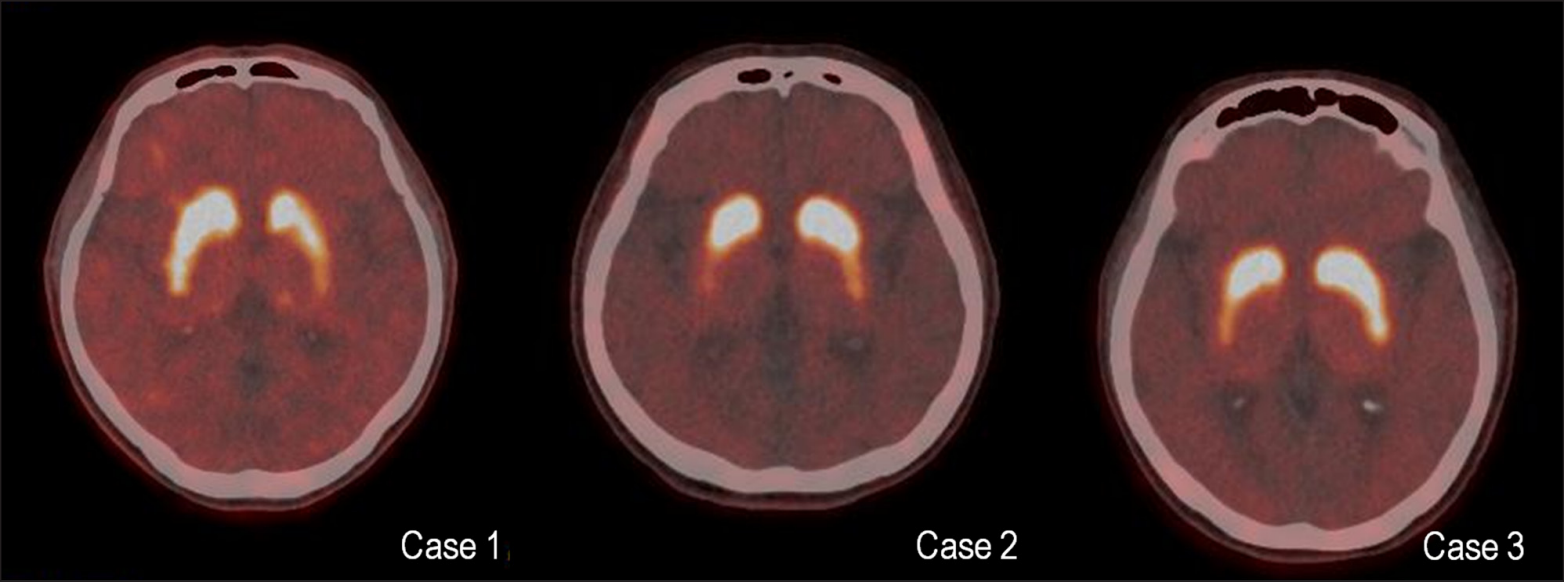
^18^F-FP-CIT PET revealed asymmetrically decreased uptake in the posterior putamen in all three cases. ^18^F-FP-CIT; ^18^F-fluorinated N-3-fluoropropyl-2-beta-carboxymethoxy-3-beta-(4-iodophenyl) nortropane, PET; positron emission tomography.

The three patients underwent dopamine transporter imaging using ^18^F-fluorinated N-3-fluoropropyl-2-beta-carboxymethoxy-3-beta-(4-iodophenyl) nortropane positron emission tomography, which revealed asymmetric decrease in the posterior putaminal uptakes in all three cases (Figure 1).

## Discussion

We report three PD cases presenting with unilateral resting tremors in a thigh muscle. The unique feature of this tremor was that it started explicitly on a semi-supine position and evolved into a unanimous appearance appearing in the sitting cross-legged position. The frequencies of the thigh tremor in our patients were all in a similar range of classic resting tremor in PD (4–7Hz). The response to dopaminergic medications of this thigh tremor was variable from excellent in the 2^nd^ case to the fair in the 3^rd^ case.

Rest tremor examination is usually performed while patients sit, stand, walk, and lie down with commanding explicit maneuvers. The typical resting tremor in the legs is usually provoked in the distal part while the patient is seated with their feet free from the floor or lying down [5] with performing a repetitive movement of other body parts as a way of distraction. The unilateral leg tremor was easily diagnosed in our first case because the leg tremor also appeared in a supine position. However, the 2^nd^ and 3^rd^ cases might have been missed unless we appropriately examined them in a semi-supine or cross-legs position. Our atypical thigh tremor cases could extend our knowledge on the resting tremor appearing in the leg in humans. The dopaminergic loss in the dorsolateral striatum somatotopically corresponds to the lower extremities, but it can not precisely explain why the proximal leg involvement was more prominent than the distal part in these patients. When resting tremor restrictively involves a proximal thigh muscle, it is necessary to evaluate patients in more diverse positions. In particular, the resting thigh tremor while sitting with legs crossed can draw physicians’ attention due to a culturally distinct characteristic in Korean people who usually sit with this position in routine daily activities.

In conclusion, even if the patient showed a different pattern of the resting tremor compared with typical PD patients, the possibility of PD should be kept in mind with isolated unilateral resting tremor, especially in a thigh muscle.
